# Spontaneous mesenteric hemorrhage following a sneezing episode associated with an intestinal pseudodiverticulum: a case report

**DOI:** 10.1097/RC9.0000000000000609

**Published:** 2026-06-25

**Authors:** Melissa Lagger, Almir Miftaroski, Gorun Ilanjian, Lucien Widmer, François Pugin

**Affiliations:** a Department of Surgery, Fribourg Cantonal Hospital, Fribourg, Switzerland; b Faculty of Science and Medicine, University of Fribourgeois, Fribourg, Switzerland; c Department of Radiology, Fribourg Cantonal Hospital, Fribourg, Switzerland

**Keywords:** case report, intestinal pseudodiverticulum, mesenteric hemorrhage, mesenteric hematoma, spontaneous intra-abdominal bleeding

## Abstract

**Introduction:**

Spontaneous mesenteric hemorrhage (SMH) is a rare and potentially life-threatening cause of acute abdominal pain. It is defined as bleeding into the mesentery in the absence of trauma or iatrogenic injury. Diagnosis is challenging due to nonspecific clinical presentation and imaging findings that may mimic bowel ischemia or neoplasia.

**Case presentation:**

A 45-year-old man, known for heavy smoking and untreated hypertension, presented with acute diffuse abdominal pain following a forceful sneezing episode. Physical examination revealed localized peritoneal signs. Contrast-enhanced CT demonstrated a mesenteric hemorrhage associated with moderate hemoperitoneum and focal mural hypoenhancement of an ileal loop, without active arterial extravasation. Urgent surgical exploration revealed approximately 500 mL of hemoperitoneum, a large mesenteric hematoma, and an adjacent intestinal pseudodiverticulum. Segmental small bowel resection, including the diverticulum, was performed. Histopathological analysis confirmed fresh mesenteric hemorrhage and pseudodiverticular changes without evidence of malignancy, vasculitis, or vascular malformation. The immediate postoperative course was uneventful; nevertheless, the patient developed a symptomatic incisional hernia.

**Discussion:**

SMH is a rare cause of acute abdomen that may occur in otherwise healthy individuals. In this case, the condition was observed in temporal association with a forceful sneezing episode. The presence of an adjacent intestinal pseudodiverticulum and patient-specific vascular risk factors, including hypertension and heavy smoking, may have contributed to local vascular fragility and bleeding. However, a clear causal relationship cannot be established. Awareness of this rare entity may facilitate timely diagnosis and appropriate management.

**Conclusions:**

This case highlights a rare presentation of SMH, with multiple potential contributing factors.

## Introduction

Spontaneous mesenteric hemorrhage (SMH) is a rare and potentially life-threatening cause of acute abdominal pain, defined as bleeding into the mesentery in the absence of trauma^[^1,2^]^. Clinical presentation is nonspecific, and imaging findings can mimic intestinal ischemia or neoplasia, often leading to exploratory surgery. There are no clear guidelines on the surgical management of SMH.

HIGHLIGHTS

- Spontaneous mesenteric hematoma is a rare cause of acute abdomen.
- It may occur in patients without trauma or identifiable risk factors.
- Sneezing may act as a trigger through transient intra-abdominal pressure.
- In our case, an ileal pseudodiverticulum was found adjacent to the mesenteric hematoma and may have acted as a contributing factor.
- Surgical exploration allowed diagnosis and definitive management.

## Presentation of case

A 45-year-old Caucasian man, working in logistics and known for heavy smoking (40 cigarettes per day) and untreated hypertension, presented to the emergency department with progressively worsening diffuse abdominal pain. He had no allergies, no significant family history, and a body mass index of 30.5 kg/m^2^ (height 167 cm, weight 85 kg). Earlier that afternoon, he experienced a forceful sneeze that immediately triggered abdominal pain, which worsened over the following hours and led to an urgent presentation.

On examination, he reported severe, crampy abdominal pain (VAS 7/10) that rapidly worsened without any modifying factors. He was hemodynamically stable, with isolated hypertension (180/100 mmHg). Abdominal examination revealed localized guarding in the right hemiabdomen without rebound tenderness or distension. Bowel sounds were normal. Laboratory tests showed leucocytosis (11.7 G/L) with a normal C-reactive protein (< 5 mg/L) and hemoglobin (147 g/L).

Contrast-enhanced abdominal CT (Fig. 1) demonstrated a segmental lack of mural enhancement in a periumbilical small bowel loop, suggestive of ischemia. A 33 × 46 mm mesenteric hemorrhage with moderate hemoperitoneum was identified, without active intra-abdominal arterial extravasation. A 3-cm long blind-ending ileal loop was observed caudal to the hemorrhage.

**Figure 1.**
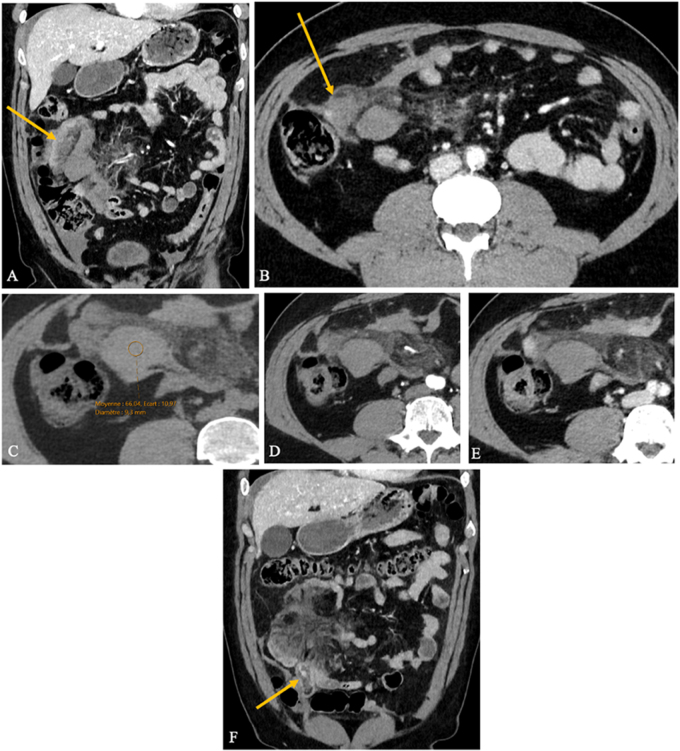
Coronal (A) and axial (B) CT scans in the portal phase show a mesenteric hematoma with surrounding mesenteric fat stranding, associated with focal wall hypoenhancement of an adjacent small bowel loop (orange arrow) and peri-hepatic and parieto-colic gutter hemoperitoneum. Axial CT scans in the native (C), arterial (D), and portal (E) phases show a contained mesenteric hematoma (spontaneous density of 66 HU), measuring 41 × 33 × 46 mm (AP × ML × CC), with no active arterial bleeding, associated with stranding of the adjacent mesenteric fat. The coronal portal phase image (F) demonstrates a blind-ending ileal loop (orange arrow) measuring approximately 3 cm, located caudally to the mesenteric hematoma, with associated stranding of the adjacent mesenteric fat.

Urgent laparoscopy was undertaken under general anesthesia by senior and junior surgeons, revealing approximately 500 mL of blood and a large mesenteric hematoma involving the ileal mesentery, with a large adjacent diverticulum (Fig. 2). Given the intraoperative concern for bowel compromise and the need for potential resection, the procedure was converted to a peri-umbilical midline laparotomy. Exploration confirmed a mesenteric tear with an extensive hematoma without bowel perforation (Fig. 2). A segmental small bowel resection, including the mesentery and diverticulum located 30 cm proximal to the ileocecal valve, was performed. Indocyanine green fluorescence angiography confirmed adequate perfusion of the remaining intestine. Intestinal continuity was restored by a hand-sewn, side-to-side anastomosis. Abdominal closure was performed using Maxon 2-0 with a small-stitch, small-bites technique, and no additional drainage was placed.

**Figure 2.**
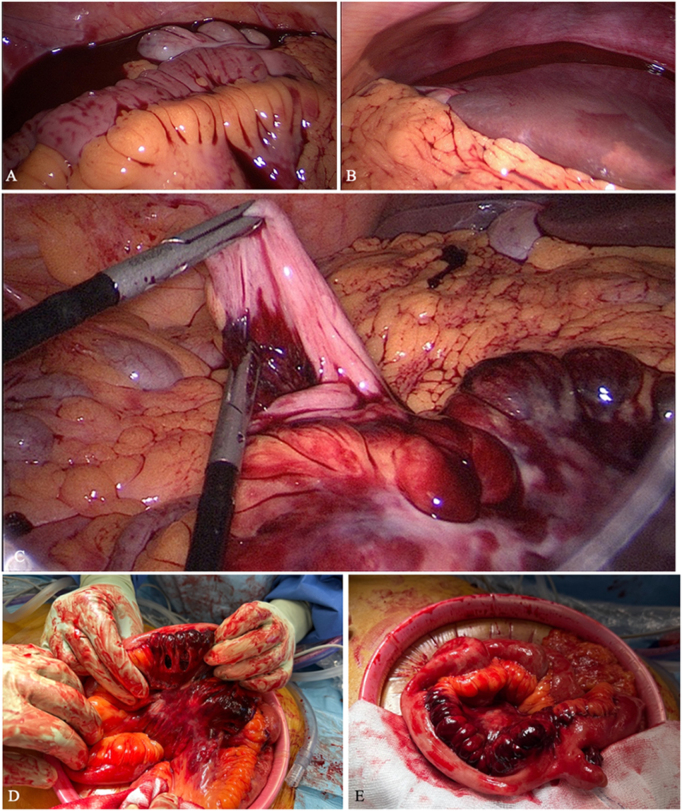
Intraoperative laparoscopy findings: (A) hemoperitoneum in the left hemiabdomen, (B) hemoperitoneum in the upper right quadrant, (C) mesenteric hematoma adjacent to an ileal diverticulum. Intraoperative findings during laparotomy: (D) mesenteric tears and hematoma, (E) ileal diverticulum and mesenteric hematoma.

Histopathological examination revealed fresh hemorrhage within peri-enteric adipose tissue, pseudo-diverticular changes without malignancy, vasculitis, or other structural vascular abnormalities (Fig. 3).

**Figure 3.**
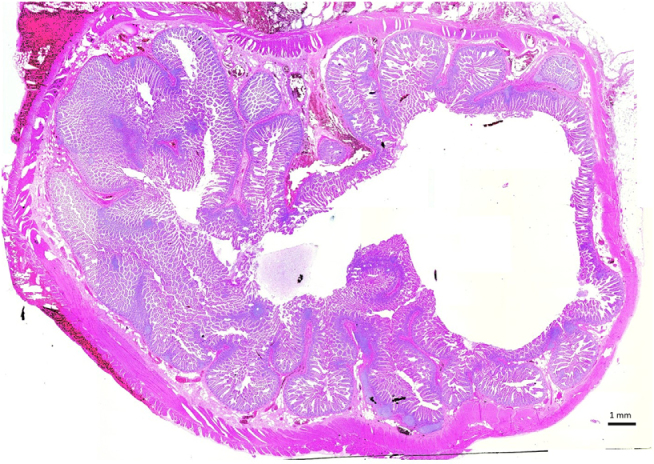
Cross-section of the pseudodiverticulum (histology). The section shows prominent plicae circulares within the intestinal lumen (H&E, 2× original magnification, composite image).

The early postoperative course was uneventful; the patient was discharged in good clinical condition on postoperative day five. At the 6-week follow-up, he reported complete resolution of abdominal symptoms, normal appetite, and a return to usual activities. During subsequent follow-up, he developed a symptomatic incisional hernia, which was surgically repaired, corresponding to a Clavien–Dindo grade IIIb complication.

## Discussion

SMH is an uncommon cause of acute abdomen, defined as bleeding into the mesentery without trauma or vascular lesion. It belongs to the broader spectrum of spontaneous intra-abdominal hemorrhage, historically termed “abdominal apoplexy,” a heterogeneous entity in which causes of mesenteric vessel rupture can be identified, including anticoagulant therapy[3], angiodysplasia of the mesenteric vessels[4], vasculitides, connective tissue disorders[5], or pancreatitis^[^6,7^]^. In contrast, true SMH refers to cases with no clear aetiology. These idiopathic mesenteric bleedings remain rare^[^8,9^]^ and diagnostically challenging, with nonspecific clinical presentations. Most reported patients present with sudden, diffuse abdominal pain of variable intensity[10].

In the present case, the patient presented with acute abdominal pain and localized peritoneal signs. The main differential diagnoses included acute intra-abdominal infections (such as appendicitis, diverticulitis, or cholecystitis), perforated hollow viscus, and acute mesenteric ischemia. Contrast-enhanced CT showed hemoperitoneum without free intraperitoneal air, making gastrointestinal perforation less likely. The absence of vascular abnormalities or arterial occlusion reduced the likelihood of aneurysmal rupture or mesenteric ischemia. The diagnosis of SMH relies primarily on contrast-enhanced CT, which typically demonstrates a spontaneous hyperattenuating mesenteric mass with surrounding fat stranding and occasionally active extravasation[11]. However, radiologic findings may mimic mesenteric tumors or bowel ischemia, and surgical exploration remains indicated when the diagnosis is uncertain or complications such as ischemia are suspected. In our patient, peritoneal signs and hemoperitoneum with suspected bowel involvement warranted urgent surgical exploration. A laparoscopic approach was initially chosen to confirm the diagnosis while minimizing invasiveness in a hemodynamically stable patient. Conversion to laparotomy was performed because of concern for bowel compromise and the need for intestinal resection.

Mesenteric hemorrhage in the absence of trauma is a rare entity. In the present case, several factors may have contributed to its aetiology: a transient increase in intra-abdominal pressure following a forceful sneeze, the presence of a pseudodiverticulum, and patient-specific vascular risk factors.

Coughing or sneezing are mechanical factors known to increase intra-abdominal pressure[12]; this could also transiently elevate mesenteric venous pressure and induce vessel rupture. Atraumatic mesenteric bleeding associated with COVID-19-related cough has been described in the literature[13].

Pseudodiverticula represent acquired outpouchings of the intestinal wall, consisting of mucosal and submucosal herniation through defects in the muscularis propria and lacking the muscular layer present in true diverticula, such as Meckel diverticulum. The absence of muscularis at the outpouching site, often where vasa recta penetrate the bowel wall, may alter local vascular architecture and increase susceptibility to vessel injury and bleeding^[^14,15^]^. Local stretching near the diverticulum may have contributed to mesenteric vessel rupture.

Hypertension has been described as a potential contributing factor to mesenteric hemorrhage^[^16,17^]^, likely through chronic vascular changes such as endothelial damage and reduced vessel elasticity. These alterations may increase susceptibility to spontaneous vessel rupture, particularly when combined with additional stressors or underlying structural weaknesses.

We report a case of SMH in a young patient without trauma, highlighting the diagnostic and therapeutic challenges in the emergency setting, supported by comprehensive clinical, radiological, and operative findings. The identification of an associated pseudodiverticulum provides an original and noteworthy aspect. However, this is a single case report, and no causal relationships can be established. The proposed mechanisms, including the role of sneezing and the pseudodiverticulum, remain speculative.

## Conclusions

SMH is a rare cause of acute abdomen occurring in the absence of identifiable risk factors. In this case, a temporal association was observed with a forceful sneeze and the presence of an adjacent intestinal pseudodiverticulum; however, these findings should be interpreted as possible contributing factors rather than established causal mechanisms.

## Data Availability

All relevant data supporting the conclusions of this article are included within the article.

## Guidelines

This case report has been prepared in line with the SCARE checklist [Supplementary Digital Content Material 1, available at: http://links.lww.com/IJSCR/A73].

## Artificial intelligence

We declare that artificial intelligence was not used in this research or manuscript development.
